# Temporal Analysis and Classification of Sensor Signals

**DOI:** 10.3390/s23063017

**Published:** 2023-03-10

**Authors:** Piotr Kosiuczenko

**Affiliations:** Institute of Information Systems, Military University of Technology, 00-908 Warsaw, Poland; pkosiuczenko@wat.edu.pl

**Keywords:** sensors, Duration Calculus, temporal logic, signal and data processing, heart rate monitoring, interval logic

## Abstract

Understanding the behaviour of sensors, and in particular, the specifications of multisensor systems, are complex problems. The variables that need to be taken into consideration include, inter alia, the application domain, the way sensors are used, and their architectures. Various models, algorithms, and technologies have been designed to achieve this goal. In this paper, a new interval logic, referred to as Duration Calculus for Functions (DC4F), is applied to precisely specify signals originating from sensors, in particular sensors and devices used in heart rhythm monitoring procedures, such as electrocardiograms. Precision is the key issue in case of safety critical system specification. DC4F is a natural extension of the well-known Duration Calculus, an interval temporal logic used for specifying the duration of a process. It is suitable for describing complex, interval-dependent behaviours. Said approach allows one to specify temporal series, describe complex interval-dependent behaviours, and evaluate the corresponding data within a unifying logical framework. The use of DC4F allows one, on the one hand, to precisely specify the behaviour of functions modelling signals generated by different sensors and devices. Such specifications can be used for classifying signals, functions, and diagrams; and for identifying normal and abnormal behaviours. On the other hand, it allows one to formulate and frame a hypothesis. This is a significant advantage over machine learning algorithms, since the latter are capable of learning different patterns but fail to allow the user to specify the behaviour of interest.

## 1. Introduction

Sensors and various types of measuring and analytical devices are becoming ever more ubiquitous due to electronic hardware being increasingly more affordable and due to the growing need for various kinds of data. Sensors detect events or changes in their environment and send the corresponding signals to other electronic devices (cf., e.g., [1,2]). Sensors are used everywhere. Various types of sensors may be distinguished: analogue sensors such as potentiometers, analogue-to-digital sensors, chemical sensors, biosensors, biochemical sensors, RIFID sensors, wearable sensors, optical image sensors, etc. The data may originate from various sources, for example, from health monitoring devices. The heterogeneity of data and their large volumes are the key problems encountered when processing the signals generated by sensors. The data generated need to be analysed on a daily basis, or even in real time if a timely response is required. Cardiovascular diseases are a major health issue and one of the primary causes of death. Today, numerous methodologies and devices are used for monitoring the heart rate and overall performance of the cardiovascular system. Various sensors can be used for this purpose (cf., e.g., [3]). However, the process of interpreting massive amounts of raw electrocardiogram (ECG) signals and data obtained from various devices is very time-consuming, if at all possible. Sensors also constitute a crucial element of the Internet of Things.

Sensor data generated by multisensor systems tend to be voluminous and heterogeneous. Before being analysed, they need to be represented in a uniform way. They may be represented in the form of temporal series, i.e., by sequences of data taken periodically, or as continuous time-dependent functions or stochastic processes. In the case of health and life critical systems, precision of specification is a key requirement.

At present, various means are commonly used to specify such heterogeneous phenomena, including diagrams, functions, tables, and textual descriptions (cf., e.g., [3]). Interrelations between different factors, regularities, and periodicity are usually described with the use of text only. However, there are problems caused by such an approach, such as imprecision and the lack of a unifying framework for specification, validation, and reasoning. Textual specifications are inherently imprecise. Instead of relying on formal semantics of comprehensive mathematical models, they use natural language with all their imprecision. Consequently, they are not capable of presenting a precise evaluation or a formal way of reasoning, meaning that their reasoning lacks due formality.

It is quite often the case that technological solutions are prepared without a proper theoretical analysis and understanding of the underlying problem. Therefore, even if those solutions turn out to work quite well, they most probably do so by accident. Hence, when new technology is developed, the problem needs to be approached anew.

One of the solutions is to use automated methods, with a particular emphasis on machine learning algorithms and methods. However, in the case of machine learning, the solutions may work quite well, but they do not contribute to the understanding of the problem or its solution. This is acceptable, for example, in the case of automated text-translation tools, but such an approach is rather problematic in the case of medical diagnosis. Most countries allow such automatic systems to be used only for assisting in the decision making process, and never as proper diagnostic systems. Technology should be based on suitable foundations, in particular, those of a mathematical and logical nature.

Logics have been developed to avoid the imprecision of natural languages. This is especially important in safety critical systems. They provide formal languages, mathematical models, and rigorous reasoning methods, and thus, deliver solutions enabling one to address the problems mentioned above. There exist various kinds of logics (see [4] for an overview). Temporal logics are aimed at expressing sequences of actions, and in general, behaviour in time. They provide temporal modalities for specifying future or past events and rules for correct reasoning about temporal properties. Different kinds of temporal logics have been developed (see [4] for an overview). It should be pointed out that logic has been traditionally a subject of intense development and study in Poland (cf., e.g., [5]). However, in general, the application of logic for the purpose of analysing and reasoning about signals has been neglected.

Of special relevance are the interval temporal logics (cf., e.g., [4,6]). They are used for specifying properties of processes concerning time intervals. Duration Calculus (DC) is a kind of interval temporal logic (cf., e.g., [7]). It is widely utilised for specifying, modelling, and reasoning about the durations of properties. It allows one to treat functions with Boolean values changing over time. It possesses an integral operator that measures the period of time over which a property remains true.

Recently, we have proposed a new interval temporal logic called Duration Calculus for Functions (DC4F) [8]. It allows one to integrate information specified by various means, such as diagrams, curves, tables, and textual descriptions in one formal framework suitable for specification, validation, and reasoning processes. DC4F is a natural extension of Duration Calculus; however, it is aimed at the specification of general integrable functions, not only Boolean-valued ones, as in the case of DC. The main idea was to apply the integral operator to general Riemann integrable functions within the well-suited logical framework of DC. As a consequence, we are able to specify not only the duration of properties, as in the case of DC, but also to specify in terms of integrals the behaviour of signals and time series over time intervals. It should be pointed out that DC4F is a conservative extension of DC; i.e., the valid formulas of DC remain valid, and the invalid formulas remain invalid.

In this paper, we use DC4F to specify various properties of signals and to describe the behaviour of sensors and sensor systems. We show how to identify that a given signal is increasing or decreasing, or that it is positive. We also explain the notions of amplitude, maximum and minimum values, and convexity. We show how to specify complex sinusoidal signals limited by functions; such curves correspond to signals generated by sensors during a stroke. It turns out that such phenomena and their interdependencies may be conveniently described using DC4F, as it offers an expressive and uniform language that may be used for describing various phenomena in a precise way. It also serves as a source of models for various types data and provides the evaluation and reasoning capabilities required.

We focus primarily on the monitoring of heart rate and of the entire cardiovascular system as such. Cardiovascular diseases are, nowadays, one of the most prevalent causes of death. On the other hand, the manner in which the cardiovascular system operates is of high interest for a number of other reasons as well, including sports achievements, general fitness, proper training, etc. Therefore, various sensors and devices are available which generate large amounts of sensor data that need to be properly analysed, interpreted, and classified in order serve as input for health and fitness assessments and for the related decision-making processes. We show how a regular electrocardiogram can be interpreted with the use of DC4F. For this purpose, we use the above-mentioned definitions of convex functions, limits, monotonicity, etc. We also argue that abnormal heartbeats can be identified in a similar manner.

It is worth mentioning that normal and abnormal cardiovascular signals are usually specified using various means, such as diagrams, textual descriptions, and tables [1,3]. These are convenient ways of expressing the forms and meanings of such signals. Unfortunately, they are imprecise and can be interpreted in various ways, creating room for misinterpretations. Additionally, they may be implemented in various incompatible ways. We show that DC4F is capable of solving these problems by providing highly precise, flexible, and expressive means for the temporal specification of signals. Furthermore, the different signal specification methods are no longer required for formal specification, since DC4F can specify the forms of signal functions and their temporal behaviours. Of course, these ways remain useful as illustrative means helping make DC4F specifications more understandable.

We also show how to use DC4F to classify signals and how to utilise the specifications in decision-making processes. The logic may be used to precisely specify when abnormal behaviour occurs, when the patient needs to be investigated further, or when a given person should reduce or intensify the level of his or her training in order to remain within the aforementioned training boundaries. Such decisions can be made on the basis of signals originating from various sensors.

This paper is organised as follows. In Section 2, we discuss the related works. Section 3 contains a brief presentation of DC4F, including its syntax and semantics, and some examples of its application. In Section 4, we define basic properties of functions, such as monotonicity and mean value. In Section 5, we discus the issue of two modes of definition: in absolute terms and relative to a measure. We define also more advanced features, such as convexity, infima, and suprema. Section 6 shows how DC4F may be applied in signal specification processes. In Section 7, we show how electrocardiograms can be precisely specified using DC4F, and how this type logic can be used for heartbeat monitoring and decision making. We conclude the paper with Section 8.

## 2. Related Works

In recent years, several sensors, multisensor systems, and devices have been developed, which generate vast amounts of data. The Internet of Things (IoT) is of particular importance here, as it enables real-time measurement and detection of various signals. This requires proper means for data processing and fusion. Data fusion consists of integrating data and knowledge originating from different sources (cf. the overview papers [9,10] and the references there). Different approaches to data fusion may be classified based on various criteria and may have the form, for instance, of data association, state estimation, or decision fusion. Data fusion has been studied in the context of temporal series as well. Various similarity measures can be used to compare such data (cf., e.g., [11,12,13,14,15]).

Cardiovascular diseases are one of the main causes of death. On the other hand, people are more and more aware of their health and use various sensors and devices to monitor their vital parameters and sport performance. Numerous portable recording devices exist, such as smart watches, bracelets, and smart shirts (cf., e.g., [1,16]).

A review of the latest developments in the monitoring of various physiological signals with the use of sensors may be found in [1]. The authors list a variety of signals used to monitor the cardiovascular system, including pulse signals, electrocardiograms (ECG), phonocardiograms (PCG), seismocardiograms (SCG), ballistocardiograms (BCG), and apexcardiograms (ACG).

ECG is the standard means for detecting cardiovascular abnormalities. The problem is that heart problems may not always be observed during a standard recording session in a hospital or clinic. To detect such problems, long term, continuous ECG monitoring of the patient’s heart is required. The development of new sensing technologies has made that possible. An accurate anomaly detection algorithm (RAAD) exists, reducing the rate of false ECG alarms occurring after detecting anomalies [17]. There are also methods that rely on ECG morphology features and VCG features to represent the heartbeat and to train the feature vectors [18]. The heartbeat classification methods have been validated [19].

As pointed out in [3], a 12-lead ECG signal is the standard method used for heart-rate monitoring. However, it turns out to be impractical when the patient is moving or exercising. Therefore, a need exists to reconstruct a 12-lead ECG signal from three lead signals and signals originating from other devices (see [3] and the references there). The results showed that the synthesised ECG signal was distorted to some extent. Thus, neural networks were used to extract the 12-lead ECG output. The standard approach to synthesising a 12-lead ECG signal from its three lead components is known as the Adaptive Region Segmentation-based Piece-wise Linear (APSPL) method.

However, the main problem consists of analysing and interpreting massive amounts of data generated by various sensors and devices. Various logics can be used to face that challenge. However, research on such applications is still insufficient, even though some useful logics exist. Fuzzy logic (cf., e.g., [20]) is a kind of many-valued logic with truth values ranging between 0 and 1. It is meant to specify properties which partially hold and reason about imprecise and non-numerical information. Temporal logics (TL) are intended to specify time dependent behaviours (cf., e.g., [4]). Their languages provide temporal modalities for specifying past or future events, and their temporal relations—e.g., property A will occur in the future, property A must always hold, property A holds until property B starts to hold, etc. The reasoning system provided by TL was used to prove that conclusions drawn from the assumptions made are correct, that implementations satisfy the corresponding specifications, etc.

Various types of temporal logics have been proposed (see [4,21] for the overview). Linear temporal Logic (LTL) specifies behaviours in terms of linear sequences of events. It provides temporal operators such as until, always, and sometime referring to linear time. Computation tree logic (CTL) specifies temporal possibilities in terms of tree-like branching time structures instead of linear sequences. Metric Temporal Logic (MTL) is a linear time temporal logic which allows one to measure the lengths of time intervals. It was used to synthesise controllers and applied in areas such as robotics (see, e.g, [22] and references there). An approach to signal specification is offered by the signal temporal logic (STL) (see, e.g., [23]). It is a linear-time temporal logic aimed at the specification of time-dependent signals originating from continuous- or hybrid-state dynamical systems (see [23] and references there). It provides a quantitative measure of property satisfaction. Interval temporal logic (ITL) (cf., e.g., [4,6]) specifies behaviours in terms of time intervals. It allows one to express the fact that a property holds within a given time interval, which is then followed by another time interval for which another property holds.

Duration Calculus (DC) can be seen as a quantitative extension of ITL. It is a widely used and throughout studied type of ITL (cf., e.g., [7]). In addition to the above-mentioned features of ITL, DC contains an integral operator that allows one to express durations of properties in a quantitative manner. DC can specify time constraints of dynamic systems. Diverse methods are utilised, including integer linear problem solving and optimisation problem solving (cf. [24]). Its range of applications vary from real-time systems (cf. e.g., [25] and the references there) to hybrid systems [26]. It is used for specification of traffic systems (cf., e.g., [25]) and air traffic control systems (cf., e.g., [27]). DC is used for the synthesis of real-time system controllers (see, for example, [24]) and the references there). Such synthesised controllers are then guaranteed to monitor and control the underlying systems according to the specified requirements.

In [28], automata-based semantics of DC have been developed with the aim of specifying aspects related to covering data, real-time, and communication. The authors proposed and implemented a model-checking algorithm for a subset of DC. A wide range of tools supporting DC have been implemented (cf, e.g., [29] and the references there). A variant of DC was presented for system discounting based on the idea that an event happening earlier is more significant than similar events happening later [30]. The idea was investigated earlier in the framework of temporal logics such as LTL and CTL. The authors proved decidability in the case of model checking of timed automata and a fragment of discounted DC.

Duration Calculus, being a kind of temporal logic, is very expressive, but consequently, is not decidable. It means that there exists no algorithm checking if a given formula is always true or always false. This is a general phenomenon: high expressivity means high complexity. There exists a rather restricted form of DC, named RDC, which has a decidable inference relation [29]. In fact, model checking of DC formulas is a subject of ongoing research (cf. e.g., [31] and the references there). In paper [32], the authors proposed a method for solving reachability problems in cases of timed automata possessing multiple cost variables evolving according to specified rates.

The pioneering work on the algebraisation of DC was done in [33]. An algebraic approach based on the so-called quantales was proposed to formalise hybrid systems—in particular, hybrid automata. It was then further developed to a form of a generalised Duration Calculus. An approach based on the mathematical notion of convolution is known as well [34]. In general, such abstract forms of DC are very general but currently lack any practical applications. Thus, their significance is only theoretical.

## 3. Duration Calculus for Functions

In this section, we present the basic ideas of Duration Calculus for Functions (DC4F) (see [8] for details), an extension of Duration Calculus (DC). DC allows one to specify and reason only about durations of Boolean-valued functions. More precisely, DC allows one to express integrals of the so-called state functions, i.e., Boolean-valued functions with values 1 and 0 representing true and false, respectively. In contrary to DC, DC4F supports general Riemann integrable functions, giving it additional expressive power, which we will utilise later.

### 3.1. Basic Properties

**Figure 1:**
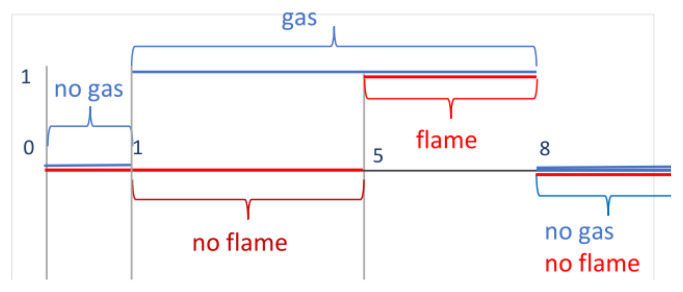
Gas- burner example.

Traditionally, Duration Calculus is illustrated with the gas-burner example (cf., e.g., [7]). Gas can be either flowing or not. It can burn or not. This behaviour can be modelled by two Boolean-valued functions: gas,flame:R→{0,1}. These functions have value 1 if gas is flowing or burning, resp., and 0 otherwise (see Figure 1). The function gas is indicated by a red line, and the function flame is indicated by a red line. In the interval [0,1], gas is neither flowing nor burning. Afterwards, it starts to leak—i.e., it is flowing but not burning. The leak state is the combination of the state gas flowing and the state gas not burning; thus, it is the conjunction gas∧¬flame. Gas leaks for 4 s, and so on. Usually for safety reasons, the duration of the gas leak has to be restricted. DC allows one to formulate corresponding safety constraints. For a time interval [a,b], we can measure the time of leak using integral of the form $\int_{a}^{b}leak$. In DC, we can formulate the requirement that, for example, gas is leaking for no more than 2 s using the formula ∫leak⩽2. Such a formula is always evaluated for an underlying interval. The fact this constraint must be satisfied for all intervals can be specified in DC using the formula □(∫leak⩽2) (below, we explain the meanings of such formulas in a detailed way). In many cases, not the leak duration matters, but the amount of gas leaked. However, in DC only Boolean-valued functions can be treated. Consequently, we cannot specify the amount of leaked gas. To specify it, one needs to integrate general functions, and thus, an extension of DC.

**Figure 2:**
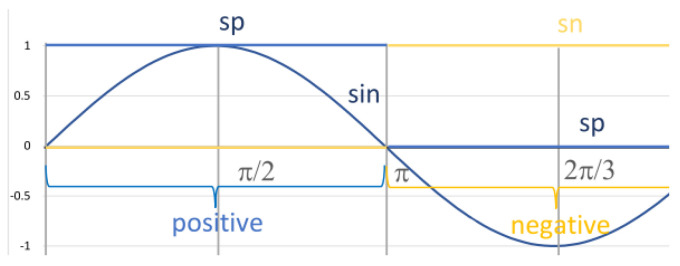
Diagram of sin and the characteristic function $1_{\left\{\;x\;|\;0\;⩽\;sin(x)\right\}}$.

We illustrate DC4F with a quite simple example of a sinusoidal signal sin(t) lasting for the time interval [0,2π] (see Figure 2). This function is not Boolean-valued and expresses certain non-binary amounts; it may express the amount of gas released. Function sin is positive on subinterval [0,π] and then negative on subinterval [π,2π]. We characterise the fact that the function is positive using the Boolean-valued function of the form $\mathrm{sp}\left(t\right)=1_{\left\{x\;|\;0⩽\sin(x)\right\}}\left(t\right)$. It returns one in the case of t∈{x|0⩽sin(x)} and zero otherwise. Thus, function sp(t) is the characteristic function of set {x|0⩽sin(x)}. Similarly, we can define function sn(t) characterising negative values. It should be reminded that we identify true with 1 and false with 0.

In DC4F, and in DC, we can measure the duration of a Boolean function, such as sp, being true. For the purpose of measuring the duration, integral operator ∫ is used. In DC4F, it merely plays the role of an integral. Thus, the duration of sp being true can be expressed as ∫sp. The semantics of the formula are defined relative to the underlying interval. Thus, if the interval is [a,b], then

(1) $$\int \;sp=\int_{a}^{b}sp\left(t\right)dt$$

Length function *ℓ* measures the length of the underlying interval. It is defined using the integration operator ∫ of DC: ℓ=∫1. The semantics of this operator are defined relative to the underlying interval. If the interval is [a,b], then $\int \;1=\int_{a}^{b}1\;dt=b-a$.

For the interval of [0,2π], the function sin is first positive and then negative. The length of the time interval, when it is positive, is equal to the length of the subinterval when it is negative. This holds for all intervals of length π. This fact may be expressed using the length function *ℓ*:

(2) ℓ=2π⇒∫sp=∫sn=π

where the function sn(t)=1−sp(t). This formula states that if the length of the interval equals 2π, then the time in which sp is true equals the time in which sn is true. We do not have to consider the fact that the function equals zero at the end of an interval, since the functions are characterised in terms of integrals, and subintervals of length zero play no role. If function sin measures the level of gas flow, then the integral ∫sin corresponds to the amount of released gas.

Using the integral operator, we can describe the basic characteristics of signals, such as mean value of a function *f* for a given interval:

(3) m=∫f/ℓ

### 3.2. The Time Domain and the Chop Operator

In this subsection, we consider the time domain of the time series and signals. We discuss the way notation can be defined and make a distinction between absolute definitions and definitions relative to the underlying measure. Finally, we define the so-called chop operator, which is crucial in both DC and DC4F.

The time domain considered here consists of non-negative real numbers, $R_{+}$. The discrete time model consisting of natural numbers N can be treated as a special case (see [8] for details). Consequently, we can handle discrete functions, or discrete time series, as if they were defined for the whole time domain.

Splitting time intervals is the fundamental operation performed by the chop operator. The chop is denoted by ${\;}^{⌢}\;$ (cf., e.g., [29]); it applies to two neighbouring intervals. For example, the sin function can be characterised as being first positive in the interval. Thus, for the interval of [0,2π], we can write $\left(\int \;sp=\ell \right){\;}^{⌢}\;\left(\int \;sn=\ell \right)$, or simply $\int \;sp=\ell {\;}^{⌢}\;\int \;sn=\ell$, meaning that the interval can be split into two parts: in the first part, sin is positive all the time, and in the other one, it is negative. The first interval is [0,π], and the other is [π,2π] (see Figure 2).

### 3.3. DC4F Formulas and Their Semantics

Every logic has an underlying syntax defining its language, and the corresponding semantics specifying the meanings of formulas. In this subsection, we briefly describe the notion of DC4F formulas. Formulas are obtained from functional expressions using logical operators. The semantics of formulas are outlined here as well (see [8] for details).

There are three main syntactic categories: functions Fn, terms Real, and formulas Fo. Elements belonging to the Fn category are interpreted as time series or signals, and more precisely as Riemann integrable functions with the $R_{+}$ domain.

Functions with finite variability in finite time are sufficient to model signals since signals are measured with finite accuracy, and at the quantum level, may have only a finite number of discontinuity points in finite time intervals. The so-called Zeno behaviours—i.e., behaviours which are characterised by infinite variability in finite time—cannot be observed. Thus, it suffices to consider functions which have finitely many discontinuity points in finite intervals. Such functions are Riemann integrable; however, the class of Riemann integrable functions is much wider. The set of functions corresponding to Fn forms a linear space; i.e., the functions may be multiplied by constants, added, and subtracted. In addition, operator f(t)∨g(t) returns the maximum of f(t) and g(t).

The category Fo consists of formulas. It plays a crucial role, as formulas are the proper objects of logical reasoning. The satisfaction of formulas is defined with respect to the underlying interval and the valuation of the variables they include. Values may be compared using operators <, =, given the underlying interval and the valuation. The result is either true or false, depending on the valuation. Alternative F∨G is satisfied if *F* or *G* is satisfied. Negation ¬F is satisfied if formula *F* is not satisfied.

In the case of chop modality, formula $F{\;}^{⌢}\;G$ is satisfied in interval *I* if there exists a real number c∈[a,b] such that *F* is satisfied in subinterval [a,c] and *G* is satisfied in subinterval [c,b]. Thus, for two formulas *F* and *G*, the $F{\;}^{⌢}\;G$ formula means that for the [a,b] interval in question, there exists a real number *c* such that a⩽c⩽b, *F* holds for subinterval [a,c] and *G* holds for subinterval [c,b]. As stated in the previous section, the fact that sin is initially positive and then negative can be specified by the formula: $\left(\int \;sp=\pi \right){\;}^{⌢}\;\left(\int \;sn=\pi \right)$. It is true for interval [0,2π], as it can be split into subintervals [0,π] and [π,2π]. We chose here x=π as the pivot point (see Figure 2). The operator ${\;}^{⌢}\;$ is associative: the form $F{\;}^{⌢}\;\left(G{\;}^{⌢}\;H\right)$ is equivalent to formula $\left(F{\;}^{⌢}\;G\right){\;}^{⌢}\;H$. This is due to the fact that these formulas concern splitting the underlying interval [a,d] into three parts, [a,b],[b,c] and [c,d], for which subformulas F, G and *H* have to hold, respectively. Consequently, the brackets can be dropped.

There is also iteration modality ${}^{*}$. It applies to a formula *F* yielding $F^{*}$. Formula $F^{*}$ is satisfied in interval *I* if there is an *n* such that we can split *I* into subintervals $\left[a,a_{1}\right]$, $\left[a_{1},a_{2}\right]$, …, $\left[a_{n-1},b\right]$, such that *F* is satisfied in each subinterval (cf. [7], Section 4 and [8]). Below, we present examples of iteration formulas.

Formulas can be quantified. Formula $\exists_{x}F$ is satisfied in interval *I* if in the time domain there exists a real value of *x* such that *F* is satisfied in *I* for this value. For example, formula $\exists_{x}x+2>25$ is satisfied since we can evaluate variable *x* by assigning it, e.g., value 24.

### 3.4. Derived Temporal Modalities

Temporal modalities play a crucial role in temporal logics. In this subsection, we present two temporal modalities which may be defined using the chop operator: the sometime and the always modality. This operator can be seen as an after modality and is characteristic of interval logics. The sometime and the always modality are used in all kinds of temporal logics (cf., e.g., [4]). However, in DC, they have a rather specific meaning: the sometime modality, denoted by *◊*, requires a property to hold for some subinterval; the always modality, denoted by □, means that a property holds for all subintervals.

Given the *G* formula and the [a,b] interval, the fact that ◊G holds for interval [a,d] means that it has a subinterval [b,c], i.e., a⩽b⩽c⩽d, such that *G* holds in [b,c]. The sometime modality may be expressed using the chop operator $true{\;}^{⌢}\;G{\;}^{⌢}\;true$, which means that we can divide the underlying interval into three subintervals and that *G* holds for the middle subinterval, whereas we do not require anything from its adjacent subintervals on the left- and right-hand sides. true means that we do not require anything in the corresponding time interval. For example, we have the following property:

(4) π<ℓ⇒◊(0<ℓ∧ℓ=∫sp)

Above formula says that if the length of the underlying interval is at least π, then it has a subinterval of non-zero length where the property sp is true all the time.

The modal formula □G expresses the fact that formula *G* holds for all subintervals of the underlying interval. For example, the formula □∫sp=ℓ means that property sp holds for all subintervals of [0,π]. The modal operator □ is defined using the sometime modality ◊: ¬◊¬G; this means that there exists no subinterval for which *G* does not hold. Vice versa, the sometime modality ◊G can be defined in terms of □: ¬□¬G. Consequently, these modalities are mutually dual. Although they are definable in terms of chop, they enhance the readability of the formulas.

Since we are often dealing with properties defined relative to the measure, it is sometimes convenient to avoid talking about what happens in intervals of length 0. Consequently, we use an abbreviation in the form of ${◊}_{+}\;\;F$ for ◊0<ℓ∧F and ${□}_{+}\;F$ for □0<ℓ⇒F. It should be noted that formula $\neg {◊}_{+}\;\neg F$ holds if and only if ${□}_{+}\;F$, which is analogous to the fact that ¬◊¬F holds if and only if □F holds.

## 4. Basic Specifications in DC4F

In this Section, we show how to define basic properties concerning behaviour in time intervals such as monotonicity of functions, approximation, and division of intervals according to phases.

### 4.1. Monotonicity

We formulate the definition of monotonicity relative to the measure. We express the fact that a function monotonically increases by requiring that for two neighbouring subintervals of the same length, the integral over the first one has a value that is smaller than or equal to the integral over the second one. This is expressed by formula Incr(f) of the form:

(5) $$\forall_{x,y}\;\;□\;\;\;\left\{\left(\ell >0\;\;\wedge \;\;\int \;f/\ell \;\;=\;\;x\right){\;}^{⌢}\;\left(\ell >0\;\;\wedge \;\;\int \;f/\ell \;\;=\;\;y\right)\;\;\Rightarrow \;\;x⩽y\right\}$$

This formula states that for a given interval and for all its adjacent subintervals, the normalised value of the integral over the one on the left-hand side is lower than or equal to the normalised value of the integral over the right one. We will abbreviate this formula as Incr(f). Analogously, the property of monotonic decrease, Decr(f), can be defined. We use capital letters in such formulas to indicate that they have a functional parameter instead of a time parameter.

### 4.2. Subdividing Intervals

Sometimes, the interplay between interval properties can be more sophisticated. The phases of positive and negative values can be divided into phases of growth and decline. In general, intervals which overlap and contain different parts of interest can be divided into smaller parts which do not overlap and components of all other units under consideration: subintervals, intervals, and groupings. Such units are commonly referred to as atomic.

**Figure 3:**
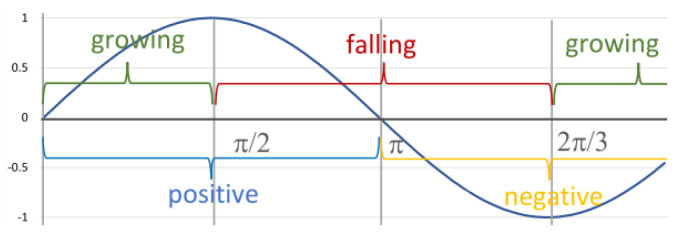
Detailed behaviour of the function sin.

We denote the phase of growth by Incr(f) (see the previous subsection), and analogously, the phase of decline by Decr (see Figure 3). Using these terms, we can specify the behaviour of the sin function for interval [0,2π] as growing and then falling:

$$Incr\left(sin\right){\;}^{⌢}\;Decr\left(sin\right){\;}^{⌢}\;Incr\left(sin\right)$$

Similarly, we may define the phases of positive and negative values of functions *f* as Pos(f) and Neg(f), respectively. We can write $Pos\left(sin\right){\;}^{⌢}\;Neg\left(sin\right)$. This formula holds for interval [0,2π], since the subformulas hold for intervals [0,π] and [π,2π], respectively (cf., Section 3.1).

Such formulas can be combined into one:

$$\left(Pos\left(sin\right){\;}^{⌢}\;Neg\left(sin\right)\right)\wedge \left(Incr\left(sin\right){\;}^{⌢}\;Decr\left(sin\right){\;}^{⌢}\;Incr\left(sin\right)\right)$$

In this case, the phases of negative and positive values can be subdivided into phases of growth and decline, which is not always the case. Thus, in this case, we can strengthen the previous formula and write

(6) $$\left(Pos\left(sin\right)\wedge \left(Incr\left(sin\right){\;}^{⌢}\;Decr\left(sin\right)\right)\right){\;}^{⌢}\;\left(Neg\left(sin\right)\wedge \left(Decr\left(sin\right){\;}^{⌢}\;Incr\left(sin\right)\right)\right)$$

This formula holds for interval [0,2π], since it can be decomposed into four subintervals: [0,π/2], [π/2,π], etc. In the first one, sin is positive and growing. In the second one, it is still positive but falling, etc.

### 4.3. Approximation

We can express the fact that function *g* is an upper approximation of function *f* by demanding that for each subinterval of a given interval, the integral of *f* is smaller than or equal to the integral of *g*:

(7) $${□}_{+}\int \;f\;\;⩽\int \;g$$

In this way, we may express the fact that function *f* has values below or above a certain fixed limit of *y*.

The property that function *h* is above fixed limit *y* for some non-zero time can be expressed by requiring that for some non-zero subinterval and all its sub-subintervals, the average value of *h* is above *y*:

(8) $${◊}_{+}\;\;\;{□}_{+}\;\;y⩽\int \;\frac{h}{\ell }\;\;$$

The amplitude of a periodic function is the largest excursion from the equilibrium position. This notion applies to oscillatory motion and wave-like functions. In the case of sin, it equals 1. The peak-to-peak amplitude is the difference between the maximum value and minimum value. If a function is for some time above value *y*, for some time below value *x*, and y−x=d, then its peak-to-peak amplitude of a function *f* is at least *d*. This property can be expressed as follows:

(9) $$\exists_{x,y}\left({◊}_{+}{□}_{+}\;\int \;\frac{h}{\ell }\;\;⩽x\right)\;\;\;\wedge \;\;\;\;\left({◊}_{+}\;\;{□}_{+}\;\;y⩽\int \;\frac{h}{\ell }\right)\;\;\;\wedge \;\;\;\;y-x=d$$

**Figure 4:**
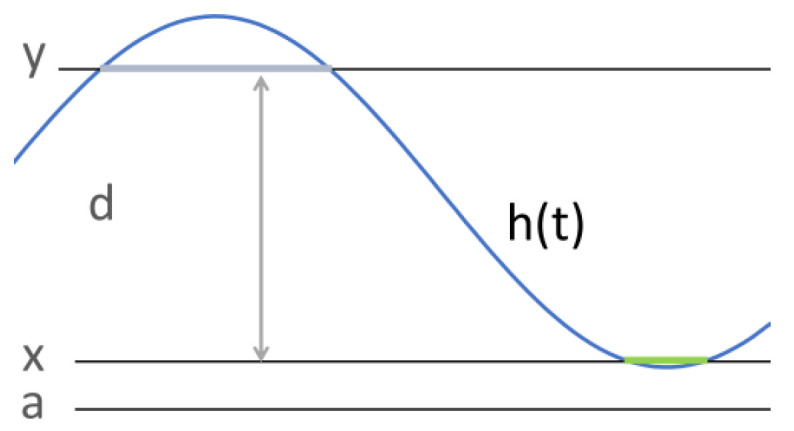
Constant limits and amplitude.

Formula (9) utilises Formula (8). It says that the given interval includes two subintervals of non-zero length. Moreover, the mean value of *h* for the first subinterval is smaller than or equal to *x*, as expressed by Formula (8). Analogously, the mean value of *h* for the second subinterval is above *y*. This formula requires, in addition, that the difference y−x be equal to *d*. Figure 4 illustrates this situation. The intervals corresponding to the parts of diagrams of *h* located below *x* and above *y* are indicated by bold lines.

## 5. Advanced Feature Specification in DC4F

In this Section, we present more advanced definitions. We define properties relevant for signal specification, such as convexity, suprema, and infima. We show that definitions can be formulated either in absolute terms or relative to a measure. Here, the original contribution of this paper starts, whereas in the previous three sections, we mainly recapitulated the basics of DC4F presented in the paper [8].

### 5.1. Pointwise Values

Access to function’s values is not directly provided in DC4F. However, if identity Id(t)=t is present among the available functions, then we can compute the values corresponding to the beginning and the end of the underlying intervals. For an underlying interval [be,en] and be, given that en−be=ℓ, we have the equations characterising the value *m* of the integral:

(10) $$m=\int \;Id\;\;=\;\;\frac{en{\;}^{2}}{2}\;-\;\frac{be{\;}^{2}}{2}\;\;\;\;=\;\;\frac{\left(en\;+be\;)(en\;-be\;\right)}{2}\;\;=\frac{(en\;+be\;)\ell }{2}\;\;=\frac{\ell +2be\;}{2}\;\;$$

From this formula, we can compute the beginning of the underlying interval be and its end en:

(11) $$be\;=\frac{2m-l}{2},\;\;\;\;en\;=be\;+\ell$$

If Id exists among the considered functions, then we will denote the beginning of the underlying interval by be and its end en.

### 5.2. Defining Properties in DC4F

The properties defined in DC4F can be defined either in absolute terms or with the help of integrals. Properties specified using intervals do not have to hold absolutely, but they are insensitive to phenomena which hold for sets of measure 0. In the measure theory, this can be expressed by saying that they hold almost everywhere. In the probability theory, it holds almost surely; i.e., they may be false at most for sets of measure zero. The temporal properties are defined with respect to intervals and refer to properties which hold almost everywhere. In particular, the always operator □ is relative to sets of full measure. It should be pointed out that in case of discrete processes, and their specification using continuous time, the difference between these two ways of definition plays no role.

Monotonicity and boundedness, just like suprema, may be considered in absolute terms and in terms that are relative to the underlying measure (see Section 4.1). In the first case, when the identity function exists and we allow the functions to assume values, then the monotonicity property can be expressed in absolute terms:

(12) □f(be)⩽f(en)

### 5.3. Convex Functions

**Figure 5:**
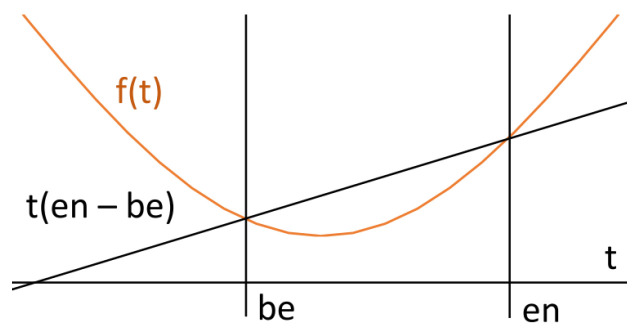
Convex function.

The existence of the identity function also allows us to define convex functions. Informally speaking, function *f* is convex if, for its two arbitrary values, a graph of *f* lies below the line connecting those values (see Figure 5). This definition can be formalised using integrals as follows:

(13) $${□}_{+}\;\;\;\int \;f\;\;⩽\;\;\int \;\left(en\;\;-\;be\;\right)\;t$$

We will denote such a formula by Convex(f). If the graph of *f* is located below the connecting line, then for the underlying interval, the integral corresponding to *f* has smaller values than the integral corresponding to the connecting line. Note that this possible if the identity function belongs to the universe of functions Fn. In a similar way, we may define the fact that the function of interest is concave, Concave(f). It should be noted that the use of integrals allows one to measure the convexity of *f* in an underlying interval as the area between the straight line t(en−be) and the curve *f*. The measure can be expressed as follows:

(14) ∫((en−be)t−f)

### 5.4. Infima and Suprema

An infimum and a supremum are not directly available in DC4F. Nonetheless, they can be defined using this logic. They can be expressed either in absolute terms or relative to a measure. In the first case, we need to use the functions be and En introduced in this paper (see Section 5.1).

The absolute infimum of *f*, for a given interval, is equal to *i* if the following formula holds:

(15) $$\begin{array}{c} \left(□\;\;i⩽f\left(be\;\right)\right)\;\;\;\wedge \;\;\forall_{0<\;\epsilon }\;\;◊\;\;f\left(be\;\right)-i⩽\epsilon , \end{array}$$

This formula says that the value of *f* for the beginning of every interval be is larger than or equal to *s*, and additionally, for every ϵ there exists a subinterval such that the corresponding mean value approximates *i* with ϵ precision.

We can also use measures and integrals and define this notion relative to a measure. Thus, if here and there, function *f* assumes erratic values, these values can be neglected, not in the absolute sense, but relatively to a measure, since values for sets of measure zero do not play any role. Note that all finite sets have measure zero. Thus, *i* is the infimum if for all its subintervals of nonzero length, the mean value is above *i*, and moreover, *i* can be approximated with arbitrary precision (the division is well defined, since only intervals of non-zero length are considered):

(16) $$\begin{array}{c} \left({□}_{+}\;\;i\;⩽\;\;\int \;\;\frac{f}{\ell }\right)\;\;\;\wedge \;\;\forall_{0<\;\epsilon }\;\;{◊}_{+}\;\;\int \;\;\frac{f}{\ell }-i\;\;⩽\;\epsilon \end{array}$$

This kind of infimum will be denoted by $Inf_{rm}\left(f\right)$. Similarly, we can define supremum $Sup_{rm}\left(f\right)$.

## 6. Signal Specification

There is a rich and advanced theory of signal processing (cf., e.g., [35]). To demonstrate the applicability of DC4F, in this section, we define several basic notions concerning signals, such as periodicity, mean value, energy, and mean power. We define the notion of periodic signals and show how to specify complex, stroke-like signals.

### 6.1. Mean Value, Energy, and Power

In this subsection, we define in DC4F notions such as the mean value, energy, and power of signals.

Above, we have defined mean values for finite intervals (see Formula (3)). However, for signals of infinite duration, this definition is not sufficient. In this case, the mean is defined as

$$\lim_{T\to \infty }\frac{1}{2T}\int_{-T}^{T}f\left(t\right)dt$$

This formula cannot be directly expressed in DC4F for two reasons: the semantics of DC4F, and DC, concerns intervals of finite length, and moreover, there is no notion of limits. The semantics of DC, and also of DC4F, is defined for finite intervals. However, if an infinite interval is allowed, then we can specify the mean value conditionally, using means of DC4F. The following formula says that if an interval is arbitrarily long, then it contains subintervals long enough to approximate the mean value *m* with arbitrary precision:

(17) $$\begin{array}{c} \left(\forall_{0<x}\;\;◊\;\;\;be\;=-x\wedge en\;=x\right)\;\;\;\Rightarrow \;\;\;\forall_{0<\;\epsilon }\exists_{0<T}\;\;◊\;\;be\;=-T\wedge en\;=T\wedge \left|\;m-\frac{1}{T}\int \;\;f\;\right|\;\;⩽\epsilon \; \end{array}$$

The antecedent of the implication requires that the underlying interval has subintervals of the form [−T,T] for arbitrary *T*. This can be satisfied only for the interval [−∞,∞]. If this property holds, then it is required that for every ϵ there exists a δ such that the mean value *m* is approximated up to ϵ for subintervals of the form [−δ,δ]. It should be noted that we do not use here the absolute value, as it can be expressed by conjunction: |x|⩽ϵ is equivalent to x⩽a∧−x⩽ϵ.

Energy *e* of signal *f* is defined using square $f^{2}$. The square does not have to belong to the universum of functions Fn. However, if this is the case, then we can define it like the case presented above, in the following manner:

(18) $$\begin{array}{c} \left(\forall_{0<x}\;\;◊\;\;\;be\;=-x\wedge en\;=x\right)\;\;\;\Rightarrow \;\;\;\forall_{0<\;\epsilon }\exists_{0<T}\;\;◊\;\;be\;=-T\wedge en\;=T\wedge \;\left|e-\;\int \;\;f^{2}\;\right|\;\;⩽\;\epsilon \; \end{array}$$

This formula says that if the underlying interval has the form [−∞,∞], then *e* can be approximated with an arbitrary precision by integral $\int \;\;f^{2}$. Note that the function is integrated over an interval of sufficient length.

Similarly, in the case of a signal *f*, we can specify its mean power and its root mean:

(19) $$\lim_{T\to \infty }\frac{1}{2T}\int_{-T}^{T}f^{2}\left(x\right)dx,\;\;\;\lim_{T\to \infty }\sqrt{\frac{1}{2T}\int_{-T}^{T}f^{2}\left(x\right)dx}$$

The second definition is possible provided that the square $f^{2}$ belongs to the universum of functions Fn and we allow the square function $\sqrt{x}$, among other functions, on real numbers.

### 6.2. Periodic Signals

In this subsection, we show how to specify periodicity of signals in DC4F. A periodic signal is a signal which is periodically repeated. More precisely, signal *f* is periodic, with period *T*, if f(x)=f(x+T). In this case, 1/T is the frequency of *f*. This equation cannot be expressed in DC4F directly. However, we may refer to intervals and integrals to specify this property.

Function *f* is periodic with period *T* if its behaviour is repeated after the period. This means that if for a subinterval, the value of integral equals *x*, then for the subinterval which corresponds to shifting the interval by *T*, the value of the integral is the same.

(20) $$\begin{array}{c} \forall_{x,y,z}\;\;x+y⩽T\;\wedge \;\;2T⩽\ell \;\;\wedge \;\;\;\left(\ell =x{\;}^{⌢}\;\left(\ell =y\;\wedge \;\int \;f=\;\;z\right){\;}^{⌢}\;true\right)\;\;\Rightarrow \;\;\\ \left(\ell =T+x{\;}^{⌢}\;\left(\ell =y\;\wedge \;\int \;f=\;\;z\right){\;}^{⌢}\;true\right) \end{array}$$

This formula concerns intervals of length 2T at least. If such an interval can be divided into an initial subinterval of length *x* followed by subinterval of length *y*, where the behaviour of *f* is characterised by the equation ∫f=z, then after time T+x, function *f* behaves in a similar way; i.e., in the adjacent interval of length *y*, the equation ∫f=z holds as well.

### 6.3. Stroke-like Signal Example

**Figure 6:**
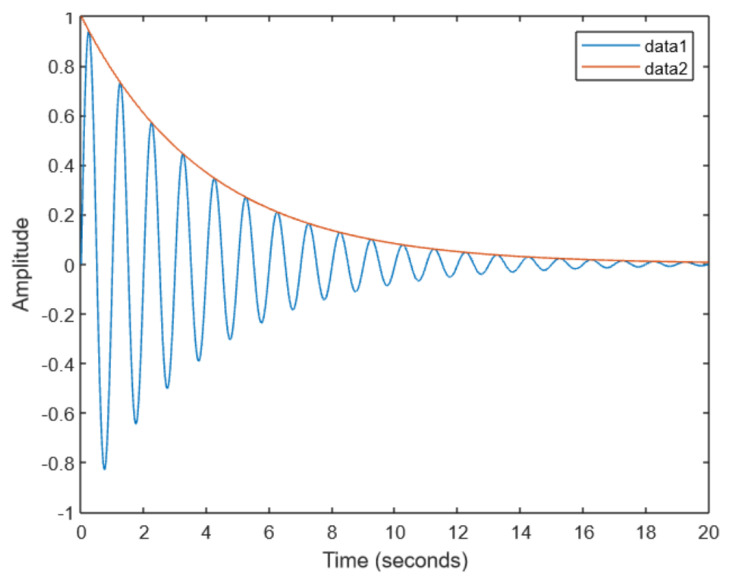
Stroke signal.

In this subsection, we present a specification example of a more complex signal, occurring in cases such as strokes. Signals of this type assume forms characterised by oscillation, and overall, a monotone decrease. An example is presented in Figure 6. Signal *f* corresponding to a stroke is plotted using the blue line (data 1). The asymptotic function *g* modelling the decline is shown using the orange line.

We specify this kind of behaviour in DC4F. The signal can be decomposed into time intervals in which its value is growing and falling. The growth of *f* can be specified by Incr(f); analogously, the decline of *f* can be specified by Decr(f). We can combine both to specify that *f* is first increasing and then decreasing, $Incr\left(f\right){\;}^{⌢}\;Decr\left(f\right)$ (see Section 4.2). This specification does not exclude arbitrary growths and decreases, and thus, functions satisfying the formula may be quite different. Therefore, it is necessary to require that the values of *f* remain below the values of *g* and that the supremum of *f* remains on the upper asymptote *g*: $Inf_{rm}\left(g-f\right)=0$. Similarly, we require that −g is the lower asymptote: $Inf_{rm}\left(f+g\right)=0$. We specify that the period of *f* is one second ℓ=1. We may combine the previous requirements together:

(21) $$\left(Incr\left(f\right){\;}^{⌢}\;Decr\left(f\right){\;}^{⌢}\;Incr\left(f\right)\right)\wedge Inf_{rm}\left(g-f\right)=0\wedge Inf_{rm}\left(f+g\right)=0\wedge \ell =1\;$$

The fact that there can be a sequence of such increases and decreases of arbitrary length can be specified using the iteration operator ${}^{*}$ (see Section 3.3). We may put all those requirements together in one formula (we revert the order of subformulas):

(22) $${\left(\ell =1\wedge Inf_{rm}\left(g-f\right)=0\wedge Inf_{rm}\left(f+g\right)=0\wedge \left(Incr\left(f\right){\;}^{⌢}\;Decr\left(f\right){\;}^{⌢}\;Incr\left(f\right)\right)\right)}^{*}\;$$

This formula says that the underlying interval can be divided into subintervals of length one such that *f* reaches *g* and −g, and in turn, the subintervals can be further divided into three parts where *f* increases, *f* decreases, and *f* increases again.

## 7. Heart-Rate Monitoring

Today, various sensors and devices exist that are used to monitor the cardiovascular system. They produce large amounts of sensor data which need to be properly analysed, interpreted, and classified in order to be used for health and fitness assessment, and for decision making. We show how the normal form electrocardiogram (ECG) can be specified in DC4F. We use the definition of convex functions, limits, monotonicity, etc. In the case of health and life-critical system specifications, precision is a key requirement. The definitions below refer to a well-defined mathematical model, and thus, are very precise. This would be very hard to achieve, if possible at all, using natural language due to its inherent imprecision.

### 7.1. Electrocardiograms

In this subsection, we informally describe ECG signals the way it is usually done in the literature (cf., e.g., [1,3]). We utilise the standard means used for this purpose: diagrams and informal descriptions. Actually, tables and formulas are sometimes utilised as well; cf., [1,3]. We specify exemplary durations of time intervals; complete specifications can be found in the standard literature (cf., e.g., [36]).

An ECG shows waves: P-waves, Q-waves, R-waves, S-waves, T-waves, and sometimes, U-waves. T-waves are normally rounded; they rise more slowly than they fall. The waves are characterised by amplitude, duration, suprema, and maxima. The events correspond commonly to individual peaks and bottoms, starts, and ends of the waves. The amplitude of a P-wave usually does not exceed 2.5 squares of the standard square-grid used in ECG. Normally, the duration of the P-wave does not exceed 120 ms. Time intervals between two specific ECG events are also a key characteristic of ECG. The intervals specify the shape of the corresponding curve, the extreme values, and the time between the initial and final event.

The intervals include the so-called PR interval between the peak of the P-wave and the peak of the R-wave, the QRS interval, the RR interval, the PP interval, and the QT interval. Intervals are usually between 120 and 200 ms. The duration of intervals is one of the key characteristics. For example, the normal duration of the QT interval is a bit shorter than or equal to 400 ms in the case of men and shorter than or equal to 440 ms in the case of women. The normal duration the PR interval is between 120 and 200 ms. There exist various typical abnormalities of the PR interval, including interval prolongation, shortening, and interbeat change.

ECG-waves can be joined into one grouping. The QRS interval is such a major grouping. The QRS grouping consists of the Q-, R-, and S-waves and represents the so-called ventricular depolarisation. It has a normal duration of between 80 and 100 ms. A duration between 100 and 120 s is considered slightly extended. A duration greater than 120 ms is considered abnormal. After the QRS grouping, there normally appears the T-wave, indicating the so-called ventricular repolarisation. A normal T-wave is partly upright and partly inverted, which parts are less than 5 and 10 mm long, respectively. A normal ST segment is usually flat at the baseline, and neither positive nor negative. However, it may be slightly elevated over normal conditions or lowered. The elevation or lowering is usually less than 1 mm. Pathological conditions, such as myocardial infarction, exhibit characteristic abnormal deviations located in the ST segment. Different patterns of ECG can be seen in patients under different conditions: sitting idle, asleep, running, or after a phase of abnormal heartbeat.

### 7.2. Application of DC4F to ECG Specification

In the previous subsection, we specified normal ECG signals using such informal means as diagrams and textual descriptions. These means are very useful to convey the basic meaning, but lack precision and mutual integration. Using means introduced in Section 5 and Section 6, we demonstrate that ECG can be very precisely specified in DC4F. This does not imply that informal means are out of place. In fact, they are very useful in conveying intuitive meaning, and this is their proper role. Nonetheless, it is worth noting the flexibility of DC4F in defining constraints concerning time intervals, subintervals, and groupings.

**Figure 7:**
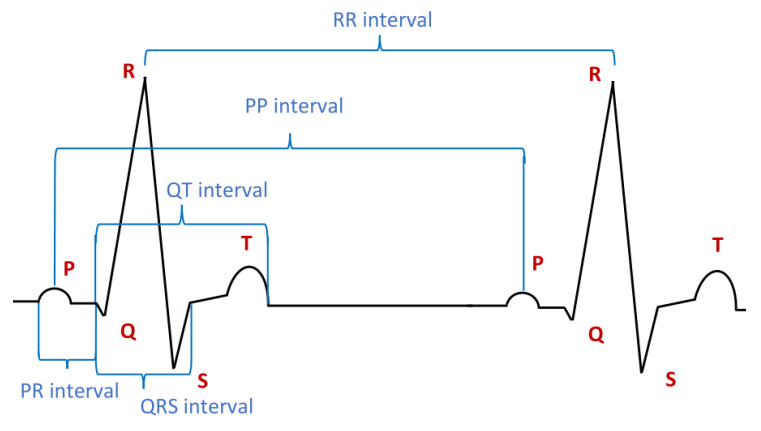
A normal ECG.

**Figure 8:**
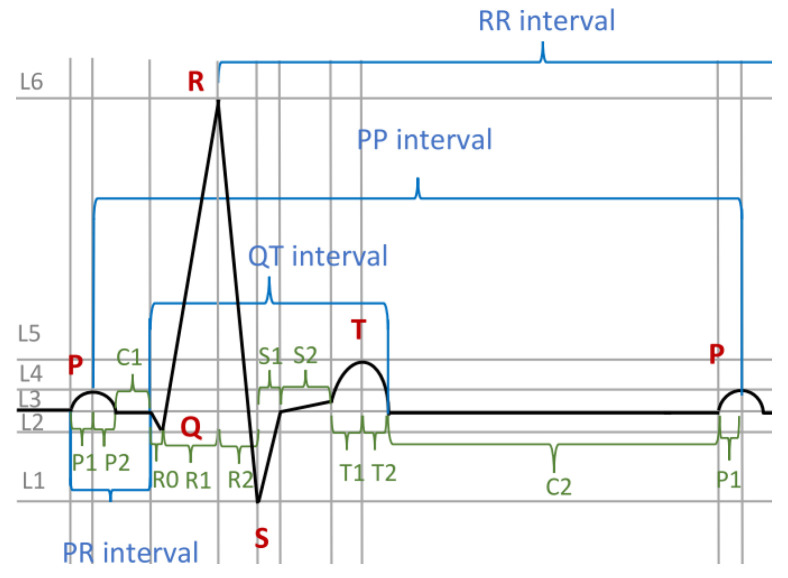
The ECG with interval decomposition.

The intervals considered before contain waves and groupings, and additionally, some of them overlap. Thus, we have to divide them into non-overlapping parts, as was done in Section 4.2. Thus, we may divide the PP interval (see Figure 7) into subintervals which are atomic: P2,C1,R0,R1,R2,S1,S2,T1,T2,C2,P1 (see Figure 8). Having the PP interval appropriately divided, we can characterise the atomic subintervals one by one. Then, we can specify the suprema and infima of waves. We can specify their durations, amplitudes, shapes, etc.

Let function *f* depict the time course of the heartbeat. For interval P2, we can characterise the corresponding behaviour in DC4F in the following manner:

(23) $$Decr\left(f\right)\wedge Convex\left(f\right)\wedge Inf_{rm}\left(f\right)=L3\wedge Sup_{rm}\left(f\right)=L4$$

as the function is convex and it decreases within the specified limits; we call this formula FP2. The DC4F formula characterising interval C1 may have the form of $Inf_{rm}\left(f\right)=L3\wedge Sup_{rm}\left(f\right)=L3$, as the function is supposed to be constant within the interval; we call this formula FC1. The DC4F formula characterising interval R0 may have the form of $Decr\left(f\right)\wedge Inf_{rm}\left(f\right)=L2\wedge Sup_{rm}\left(f\right)=L3$, as the function decreases within the interval. We call this formula FR0. Similarly, we can characterise the behaviour of *f* in other intervals.

Thus, we can join the specifications of *f* concerning interval PP in one DC4F formula:

(24) $$FP2{\;}^{⌢}\;FC1{\;}^{⌢}\;FQ1{\;}^{⌢}\;FR0{\;}^{⌢}\;FR1{\;}^{⌢}\;FR2{\;}^{⌢}\;\cdots {\;}^{⌢}\;FP1$$

It should be noted that there are time limits for the waves and the composed intervals shown in Figure 7, such as the P-wave and QT interval. However, those limits do not apply directly to the atomic subintervals shown in Figure 8. We can nevertheless specify those overall time limits by extending the above formula to formula NHRE:

(25) $$\begin{array}{c} (\left(FP2\wedge \ell =lP2\right){\;}^{⌢}\;\left(FC1\wedge \ell =lC1\right){\;}^{⌢}\;\cdots {\;}^{⌢}\;\left(FP1\wedge \ell =lP1\right))\;\;\wedge \\ lP1+lP2=120\;\wedge 80⩽lR0+lR1+lR2+lS1⩽100\wedge lQT=400 \end{array}$$

where lP2,lC1,lP1,lP2,lR0,lR1,⋯ are the lengths of intervals P2,C1,P1,P2,R0,R1, …, respectively. Here, we specify the time limits as listed above in the informal description. It should be noted that the time limits for the subintervals may be of no relevance, or they may be not known. The fact that we do not have to subdivide the time limits for the atomic subintervals but can specify them for their groupings, such as PP, demonstrates the flexibility of DC4F.

If the monitored person is running, then the time limits are shorter. This can be specified by adjusting the time constraints. Abnormal heartbeats can be specified along similar lines. For example, the abnormal length of QRS can be specified with the lQRS<80∨120<lQRS formula, which is just a negation of the 80⩽lQRS⩽120 formula. We can specify the abnormal shapes of *f* as negations of the formula specifying its normal shape. Sometimes, abnormal behaviours are classified into different categories corresponding to different shapes of *f* (cf., e.g., [3]). We could specify those shapes in DC4F as well.

A normal ECG is just a repetition of the normal heartbeat cycle. Thus, we can specify a normal ECG using formula $NHRE^{*}$, which means that the underlying measurement interval can be divided into subintervals in which NHRE holds. In this case, all heartbeats are normal.

### 7.3. Decision Making

In this subsection, we show how to use DC4F to specify decisions. It is possible to monitor a person during different activities, such as resting, walking, and running.

We specify different conditions using logical variable *B* with real values such as resting,walking,running,…. We take into consideration the behaviour of the monitored person and specify normal behaviour depending on his/her current activity:

(26) $$\begin{array}{c} \left(\int \;\frac{B}{\ell }\;=resting\Rightarrow NHRE^{*}\right)\;\;\wedge \left(\int \;\frac{B}{\ell }\;=walking\Rightarrow WHRE^{*}\right)\;\;\;\wedge \\ \left(\int \;\frac{B}{\ell }\;=running\Rightarrow RHRE^{*}\right) \end{array}$$

WHRE and RHRE mean a normal ECG heartbeat when a person is walking or running, respectively. Condition $\int \;\frac{B}{\ell }=resting$ means that the person is resting all the time. Thus, the mean value of *B* is resting. A similar procedure applies to other activities. Consequently, we have the corresponding *f* characteristics for different behaviours. The entire formula—let us call it *W*—means that the heart rate is normal when the person is resting or walking, but abnormal when they are running. If *W* is not satisfied, then an action can be taken. For example, we may require that if regular heartbeat is registered during a run, the person stops running within 10 s and starts walking for 10 s:

(27) $$\begin{array}{c} □\;\;\;\;(\left(\int \;\frac{B}{\ell }\;\;=running\wedge ARHR\right){\;}^{⌢}\;40⩽\ell )\;\;\;\;\;\;\;\;\;\;\;\Rightarrow \;\;\;\;\;\;\;\;\;\;\;\;\;\;\;\;\;\;\;\;\;\;\;\;\;\;\;\;\;\;\;\;\;\;\;\;\;\;\;\;\;\;\;\;\;\;\;\;\;\;\;\;\;\;\;\;\;\;\;\;\;\;\;\;\;\;\;\;\;\;\;\;\;\;\;\\ \;\;\;\;\;\;\;\;\;\;\;\;\;\;\;\left(\ell =10{\;}^{⌢}\;\left(\int \;\frac{B}{\ell }\;\;=walking\;\;\wedge \;\;30⩽\ell \right)\right){\;}^{⌢}\;true) \end{array}$$

This means that if an abnormal heartbeat is detected during a run and the interval is at least 40 s long, then within at most 10 s, the person starts walking and walks for at least 30 s. Formula AHRE means an abnormal heart rate while running. It can just have the form of negation ¬RHRE, meaning that the condition RHRE does not hold. Alternatively, AHRE can be specified in a refined way. We write similar formulas determining what needs to be done when a number of irregular heartbeats occur. We may specify those irregularities, etc.

### 7.4. Hear Attack Detection

In this subsection, we consider two characteristic patterns of ECG diagrams occurring after myocardis (heart attack; see [3,36,37]). The first one concerns R-wave progression (see Figure 7 for the R-wave). The second one concerns a specific pathological pattern of the PP intervals. Those patterns are due to the so-called myocardial ischemia (cf., e.g., [37]). Their detection strongly indicates a heart attack condition. We show how to specify them in DC4F using the above-mentioned definitions.

**Figure 9:**
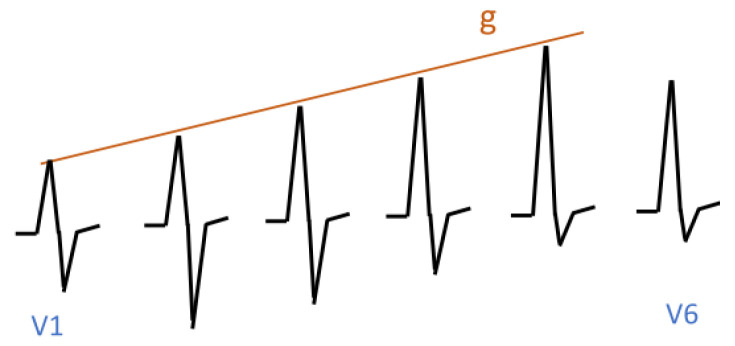
Normal R-wave progression.

**Figure 10:**
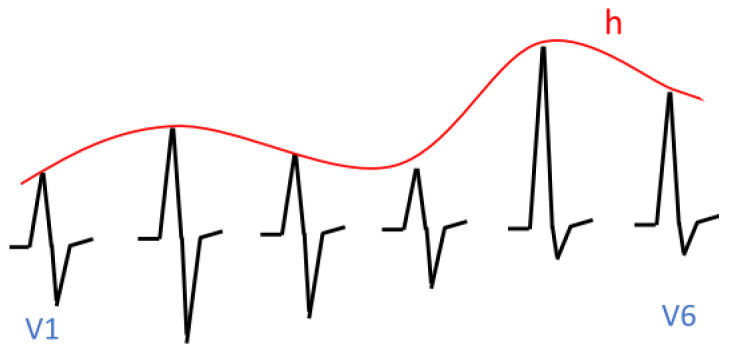
Abnormal R-wave progression.

The first example concerns the so-called R-wave progression. A normal R-wave progression is shown in Figure 9. It is characterised by the fact that in leads V1 to V5, the suprema are located on the straight line *g*. An abnormal R-wave progression is shown in Figure 10. It is characterised by the fact that in segments corresponding to leads V1 to V5, the suprema are located on the curved line *h*. Initially, this line rises, then it falls, and finally, it rises again. This is characteristic of myocardial ischemia.

In Section 5.4, we demonstrated how to specify suprema, and in Section 6.3, we demonstrated how to specify that suprema are located on a certain line (see Figure 10). In Section 7.2, we demonstrated how to specify normal ECG signals. The cases of normal R-waves can be specified analogously to the R-waves in Section 7.2. We specify the behaviour in subinterval R1: $Inf_{rm}\left(f\right)=L2\wedge Incr\left(f\right)\wedge Sup_{rm}\left(f\right)=g\left(en\;\right)$. This formula says that *f* increases, and at the end of the subinterval, it reaches the *g* line. The specification of abnormal R-waves is analogous; i.e., we replace the condition $Sup_{rm}\left(f\right)=g\left(en\;\right)$ with the condition $Sup_{rm}\left(f\right)=h\left(en\;\right)$.

**Figure 11:**
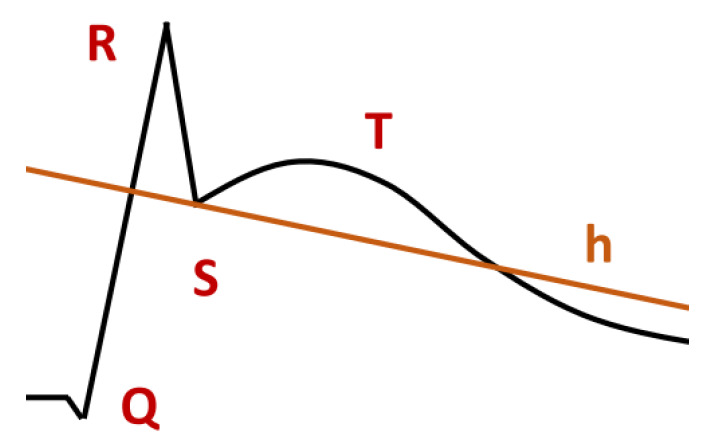
Concave ST-interval elevation.

The so-called ST-interval elevation is also characteristic of ischemia. A special case of such an elevation is the concave elevation (see Figure 11). It occurs after the R-wave and the S infimum are lifted relative to the normal ECG (see Figure 8). The following line is concave. It should be noted that, in the medical literature, the ST-interval elevation shown in Figure 11 is sometimes called convex [37]. To specify the concavity, we may use the formula Concave(f) (see Section 5.3). If needed, the concavity can be measured (see Formula (14)). The following formula says that at the beginning and at the end, the values of *f* coincide with the values of *h*, that *f* is concave, and that the measure of concavity is at least *c*:

(28) f(be)=h(be)∧Concave(f)∧f(en)=h(en)∧c⩽∫(f−h)

## 8. Evaluation and Conclusions

In this Section, we evaluate the proposed logic against similar approaches and draw conclusions concerning its potential. In the first subsection, we compare CD4F with alternative approaches. In the second subsection, we conclude the paper with the discussion of DC4F application and development possibilities.

### 8.1. Comparison with Other Approaches

Precision and correctness are key requirements in the case of health and life-critical system specification. Dedicated logics are commonly used to achieve such goals. They provide formal languages, uniform mathematical models, and reasoning rules to deal with time series and signals. Traditionally, Poland has been a place where logic has been studied and applied (see, for example, [5] for an overview). Unfortunately, in the case of signal specification, such research was lacking. In this paper, we presented the Duration Calculus for integrable Functions (DC4F) and demonstrated its applicability to signal specification. DC4F is a natural, conservative extension of Duration Calculus (DC).

DC4F logic can be best compared with other logics, rather than algorithms or tools. Logics are then used in dedicated algorithms for the specification of the phenomena of interest (cf., e.g., [22,24]). We compare DC4F with logics aimed at the specification of similar phenomena: Duration Calculus (see [7]), Metric Temporal Logic (MTL) (see [22]), Signal Temporal Logic (STL) (see [23]), and Fuzzy Logic (FL) (see [20]). These logics are closest to DC4F; thus, the questions emerge: to what extent can they be applied to specify interval-related properties of functions? How well can they specify the formal properties defined in Section 4, Section 5 and Section 6? In particular, how can they deal with the heart monitoring problem described in Section 7?

DC4F differs from DC in terms of expressivity, the scope of its applications, and of course, maturity. DC4F deals with arbitrary Riemann integrable functions. Ergo, it provides a universal model for a wide class of time series and signals. These models can be handled using means such as integral calculus and computer algebra. It allows one to specify diverse properties, relations, and dependencies in one coherent language, to model them in a uniform manner, and to reason about them.

MTL and DC are based on the idea of considering first the properties holding in a given moment of time, such as “gas is leaking” or “gas is burning”. For example, in Section 3.1, we consider if gas is flowing or not (see Figure 1), or if function sin is positive or not (see Figure 2). At any moment in time, such a property is true or false. In the case of MTL, one can then specify temporal properties using modal operators and time bounds in the form of intervals. For example, it is possible to specify that gas is leaking until gas starts to burn and no longer than, say, 2 s, or that sin is positive until sin equals zero in the interval [0,π]. However, there is no means of decomposing intervals, such as the chop modality, and no way exists for measuring the lengths of intervals for which a property holds. In the case of heart-rate monitoring (Section 7), it is necessary to decompose intervals, such as PP, QRS, QT, and PP, into subintervals in order to properly specify the forms of diagrams and to measure their lengths. Moreover, there is no way to define the iterator *.

On the contrary, DC possesses the chop operator, the iterator, and it allows one to express the lengths of intervals. For example, we can express that gas is leaking for at most 1 s by formula □(∫GL⩽1). The function GL(t) assumes the value of one if gas is leaking, and zero otherwise. Nonetheless, DC, like MTL, is not capable of expressing limits of functions or other properties, such as monotonicity and convexity.

STL was introduced to deal with signals. It can be thought of as an extension of MTL with an operator allowing one to express that a given function is above a certain limit—for example, that sin is positive. This option gives some extra expressive power. However, still, one cannot specify duration of intervals, nor express properties such as monotonicity, convexity, and suprema.

Fuzzy Logic as such does not allow one to specify temporal properties, even less their duration. As a consequence, it cannot be used for this purpose. On the other hand, it is associated with various models, methods, and algorithms for the modelling and assessment of imprecisely defined properties. In particular, one can deal with a function approximation. However, such methods and algorithms could be integrated with DC and DC4F (see [8]).

Table 1 presents a bird view comparison of CD4F and the above-mentioned logics. We compare their expressibility with respect to the following criteria: the possibility to decompose intervals (abbreviated as Int. Dec.), the possibility to measure the length of intervals where a certain property holds (Length m.), the possibility of integrating (integrals), if general functions/signals can be specified (Gen. Fun.), if the time model is continuous (Cont. Time), and if model checking is supported (Model Check.). These properties proved to be essential for the specification of signals corresponding to ECG and the assessment of related medical conditions (see Section 6 and Section 7).

In Section 7.2, to specify normal ECG, we made extensive use of properties such as length measure, monotonicity, iteration, suprema, and infima. In Section 7.4), we specified abnormal ECG corresponding to ischemia; we used infima and suprema, concavity, and asymptotes. These features can be specified for general functions using integrals (see Table 1, columns 4 and 5). This is possible only in DC4F.

As mentioned in the introduction, DC4F, like DC and the first-order logic, is not decidable. This means that there exists no general algorithm allowing one to figure out if a given formula is always satisfied, or in some cases, not. Nonetheless, in the case of finite models, the validity of DC4F formulas can be validated; this is a general phenomenon concerning DC and FOL as well. It should also be noted that model checking can be done for the above-mentioned kinds of logics in the case of models of restricted size. Due to the limited accuracy of measurements, at some level, their models are discrete and finite. Model checking is possible for restricted forms of DC (cf., [31]). At present, there is no research on how to model-check DC4F models, but this issue is not relevant for the signal specification, as considered in this paper.

### 8.2. Concluding Remarks

We demonstrated how to specify in DC4F various temporal properties of signals, such as monotonicity, periodicity, boundedness, asymptotes, and oscillation, and applied them to specify ECG signals. DC4F turned out to be a suitable framework for integrating various types of data expressed in terms of time series, tables, and diagrams. Commonly, these kinds of data are specified informally, using textual descriptions, natural language, tables, and occasionally, some mathematical formulas. Of course, this manner of specifying data is imprecise and error-prone.

DC4F can be used to specify expert knowledge, frame hypotheses, and reason about and validate them. The difference between validation and reasoning is that validation is performed for a concrete data set, in a specific model, and reasoning relies entirely on the application of deduction rules. DC4F specifications can incorporate other methods, such as algorithms, trained networks, and other methods from the field of machine learning. Diverse machine learning algorithms exist (see [3] for a comprehensive survey of the existing methods). There exist methods using linear discriminant analysis for classification [38]. However, it should be stressed that machine learning algorithms do not allow one to specify properties in a conscious manner like DC4F does. The algorithms work more or less like black boxes, and users can hardly specify relevant properties, formulate observations, and frame the hypotheses on their own. In fact, this is the general problem with machine learning methods, such as neural networks, genetic algorithms, and simulated annealing. On the other hand logics, unlike learning algorithms, can express properties and expert knowledge but do not possess mechanisms for learning specific properties and relations among them.

If data (generated, for example, by multisensor systems) are provided, then DC4F formulas may be validated for them and their truthfulness checked. Consequently, DC4F provides a unifying logical framework for data integration, for reasoning, and for formulating hypotheses and their evaluation. This does not imply that the common means such as textual descriptions, tables, and formulas become obsolete. Quite the opposite is the case. They are very usable for conveying intuitions, primary ideas and specifying basic facts. In fact, this is their proper role.

DC4F proved to be well-suited to specifying the properties of signals, for example, specific patterns in ECGs. The specifications are expressed in terms of integrals and durations. This can hardly be done in other kinds of temporal logics. In the future, we envisage cooperation with medical experts: using DC4F to specify expert knowledge and to utilise it.

## Figures and Tables

**Table 1:** Expressibility comparison.

Logic	Int. Dec.	Length m.	Integrals	Gen. Fun.	Cont. Time	Model Check.
MTL	no	no	no	no	restricted	yes
STL	no	no	no	no	restricted	yes
FL	no	no	restricted	no	no	restricted
DC	yes	yes	restricted	no	yes	restricted
DC4F	yes	yes	yes	yes	yes	no
